# Production of the SARS‐CoV‐2 receptor‐binding domain in stably transformed rice plants for developing country applications

**DOI:** 10.1111/pbi.14023

**Published:** 2023-02-17

**Authors:** Andrea Saba‐Mayoral, Claudia Rosa, Guillermo Sobrino‐Mengual, Victoria Armario‐Najera, Paul Christou, Teresa Capell

**Affiliations:** ^1^ Applied Plant Biotechnology Group, Department of Plant Production and Forestry Science University of Lleida‐Agrotecnio CERCA Center Lleida Spain; ^2^ ICREA Catalan Institute for Research and Advanced Studies Barcelona Spain

**Keywords:** Oryza sativa, stable transformation, receptor‐binding domain, COVID‐19

Most of the 41 approved COVID‐19 vaccines and ~220 candidates currently undergoing clinical testing (Basta and Moodie, 2020) are designed around the SARS‐CoV‐2 spike (S) protein or the receptor‐binding domain (RBD) within it, which facilitates virus uptake by binding to angiotensin‐converting enzyme 2 (ACE2) on the cell surface. The S protein/RBD is also used as a component of diagnostic kits for the detection of antibodies and the quantification of antibody titers (Yüce *et al*., 2021). Plants have been evaluated as a large‐scale production platform for COVID‐19 vaccines and diagnostic reagents because they are more cost‐effective than fermenter platforms (Capell *et al*., 2020), making them particularly suitable for low‐ and middle‐income countries (LMICs). The transient expression has the advantage of speed and scalability and has been used to produce COVID‐19 reagents (Makatsa *et al*., 2021) and one approved vaccine (Ward *et al*., 2020). However, transgenic rice plants provide a stable resource for larger‐scale production over a longer term and have already been used to produce antiviral antibodies and lectins (Vamvaka *et al*., 2018).

As proof of concept, we transformed mature‐embryo‐derived rice callus with constructs in which the RBD transgene was expressed under the control of either the constitutive maize ubiquitin promoter and first intron (Ubi‐1) or the endosperm‐specific barley D‐hordein promoter (Hord), each paired with the rice α‐amylase signal peptide targeting the secretory pathway. We recovered 31 transgenic callus lines transformed with pUbi‐RBD and 34 transformed with pHord‐RBD. All 65 lines contained the corresponding gene of interest. The RBD content was evaluated by ELISA, using human ACE2 as the capture reagent to ensure that only the correctly folded and soluble form of the RBD was detected. Functional RBD was present in 27/31 of the pUbi‐RBD callus lines (~87%) and 22 of the positive lines (~82%) accumulated the correctly folded RBD at levels detectable by western blot. The highest yield of RBD (line 19) was 6.88 ± 1.28 μg/g fresh callus weight (fw). However, only six lines (~15%) transformed with pHord‐RBD produced a functional RBD protein and only five at levels detectable levels by western blot. The highest yield of RBD in the pHord‐RBD callus was 2.20 ± 0.9 μg/g fw (line 12), 3.12‐fold lower than the best‐performing pUbi‐RBD line (Figure 1a).

**Figure 1:**
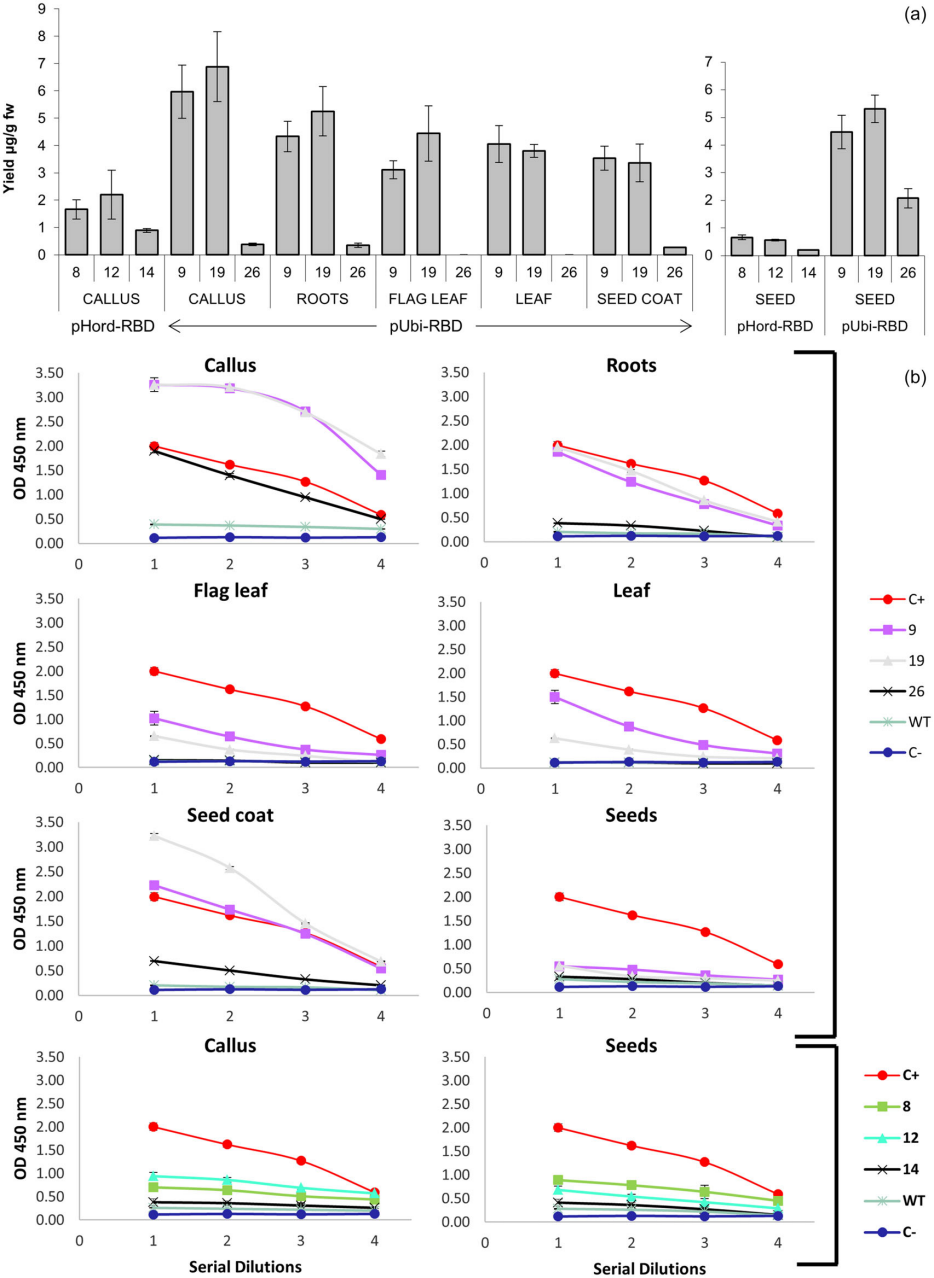
RBD yields and binding activity in crude extracts of different rice tissues. (a) The RBD yield in callus, roots, flag leaf, leaf, seed and seed coat (μg/g fresh weight) of three lines transformed with pUbi‐RBD (lines 9, 19 and 26), as determined by ELISA. The RBD yield was also determined in the callus and seeds of three lines transformed with pHord‐RBD (lines 8, 12 and 14). Proteins were extracted three times (biological replicates) and each sample was measured three times (technical replicates). Data are means of all replicates (*n* = 9) ± SE. (b) Ability of crude rice tissue extracts containing RBD to bind human ACE2 is determined by ELISA. The graphs show binding efficiency (represented by the absorbance) on the *y*‐axis over a two‐fold serial dilution range on the *x*‐axis, starting at 0.5 μg/μL total protein. ACE2 was coated onto ELISA plates (50 ng/well) and incubated with the recombinant RBD, which was detected using a primary anti‐RBD antibody and an HRP‐conjugated secondary antibody in extracts from plants transformed with pUbi‐RBD (lines 9, 19 and 26) and pHord‐RBD (lines 8, 12 and 14). Dilutions were prepared for the transgenic lines and wild‐type (WT) control. From the top left, the panels show the binding activity for the callus, roots, flag leaf, leaf, seed coat and seeds of the pUbi‐RBD lines, and the callus and seeds of the pHord‐RBD lines. Data are means ± SE of triplicates at each concentration. The positive control (C+) was SARS‐CoV‐2 (2019‐nCoV) S1 protein, prepared as two‐fold serial dilutions starting at 0.8 ng/μL. The negative control (C^−^) was 1% BSA in PBST, also prepared as two‐fold serial dilutions. OD = optical density at 450 nm.

We regenerated 15 independent T0 transgenic rice plants containing pUbi‐RBD and 14 containing pHord‐RBD. Three independent T0 plants representing pUbi‐RBD (lines 9, 19 and 26) and three representing pHord‐RBD (lines 8, 12 and 14) were taken to maturity, and all six were shown to contain the gene of interest by PCR. Representative T1 seed extracts were tested by ELISA as described above. The maximum quantity of soluble and correctly folded RBD in the pUbi‐RBD seeds was 5.31 ± 0.50 μg/g dw in plant 19, whereas the maximum in the pHord‐RBD seed extracts was 0.66 ± 0.08 μg/g dw in plant 8 (Figure 1a). RDB was also detected in the leaves of nine pUbi‐RBD plants (60%) and the maximum yield was 4.05 ± 0.67 μg/g fw in line 9 (Figure 1a). We also analysed root extracts from plants 9, 19 and 26, and the highest yield was 5.25 ± 0.90 μg/g fw in line 19. RBD also accumulated in the flag leaves of plants 9 (3.11 ± 0.33 μg/g^−1^ fw) and 19 (4.44 ± 1.01 μg/g^−1^ fw) but not plant 26, and accumulated in the seed coat of all three plants, with the highest level in plant 9 (3.54 ± 0.44 μg/g fw). Western blot analysis revealed multiple bands in the flag leaf, leaf, seed coat and seed that reveal differing glycosylation profiles in those tissues, based on the presence of two *N*‐linked glycosylation sites (N331 and N343) in our construct (Figure S1 shows line 9 as a representative example). Finally, we evaluated the ability of seed‐derived RBD from all six lines to bind ACE2, confirming specific and concentration‐dependent binding in all cases (Figure 1b). These results confirmed the structural integrity of the RBD and indicated that rice seeds are suitable for the development of a scalable production platform.

To the best of our knowledge, this is the first report in which any part of the SARS‐CoV‐2 S protein has been expressed in transgenic plants. Several reports describe the production of RBD by transient expression in *N. benthamiana* leaves with yields ranging from 2 to 4 μg/g leaf fw for an RBD construct (like ours) spanning residues 319–541 (Diego‐Martin *et al*., 2020) to 117 ± 41 μg/g leaf fw for a truncated construct (residues 319–533) lacking the cysteine residue at position 538 (Shin *et al*., 2021). The yields vary according to the length of the RBD sequence, the presence of other constructs, the gene transfer method, the number of cysteine residues and the subcellular targeting strategy. Our maximum yields of 6.88 ± 1.28 μg/g fw in the callus of line 19 (pUbi‐RBD) and 4.05 ± 0.67 μg/g fw in the leaves of line 9 (pUbi‐RBD) compare favourably with earlier studies expressing the same RBD variant (residues 319–541) in *N. benthamiana* leaves (Diego‐Martin *et al*., 2020; Maharjan and Choe, 2021; Shin *et al*., 2021). The maximum binding activity was detected in callus tissue, followed by the seed coat and roots of both lines. Although the binding of RBD in seeds was lower than in other tissues, possibly reflecting the presence of seed‐specific RBD glycans that interfere with binding, or competition with rice storage proteins, the yields were more than sufficient to confirm proof of concept for the development of seeds as a production and storage platform in LMICs. Current yields of ~5 mg/kg dw would be equivalent to 50 g/ha assuming a biomass yield of 10 t/ha, which would be sufficient for 1400 vaccine doses of 25 μg each, assuming 70% recovery (Lico *et al*., 2020). Using construct optimization and breeding to improve the yield 10‐fold to 50 mg/kg would produce ~500 g/ha, equivalent to 14 000 vaccine doses at the same efficiency of recovery.

In conclusion, we have expressed the SARS‐CoV‐2 RBD in a range of tissues, including seeds, in stably transformed rice plants. We evaluated the constitutive and seed‐specific expression of the RBD and found that the former achieved the highest yields in all tissues, including callus and seeds (where the endosperm‐specific promoter is also active). The next step to advance this proof of concept is to express the protein with a cleavable purification tag to simplify protein isolation, allowing the purified native protein to be released for functional evaluation. Based on the maximum yields we achieved using each construct, we conclude that the constitutive promoter was three times more active in callus and >40 times more active in the seeds, but that the RBD yield in seeds was still more than sufficient for the development of a production platform in LMIC settings, where the lack of resources makes it more difficult to access vaccines and reagents produced using traditional fermenter‐based platforms.

## Conflict of interest

The authors declare no conflict of interest.

## Author contributions

PC and TC conceived the project, PC and TC designed the experiments, ASM, CR, GSM, VAN and TC performed the experiments, ASM, GSM, VAN and TC analysed the data, ASM, TC and PC wrote the manuscript.

## Supporting information


**Figure S1** Western blot analysis of crude extracts from different tissues of line 9 expressing the pUbi‐RBD construct. The samples were probed with a primary anti‐RBD antibody and detected with an AP‐conjugated secondary antibody. All lanes contain 50 μg of total protein. The Ponceau stained gel is shown as a loading control, with size markers in the leftmost lane (L, ladder). The expected size of RBD is 23 kDa. The three observed bands at ~23 kDa (arrow) are assumed to represent variants of the protein with 0, 1 and 2 glycan acceptor sites occupied, respectively.
