# Supplementary figures and images for: Production of the SARS‐CoV‐2 receptor‐binding domain in stably transformed rice plants for developing country applications
Source: Plant Biotechnol J. 2023 Feb 17;21(6):1094–6. doi: 10.1111/pbi.14023 (PMC10214748; doi:10.1111/pbi.14023)

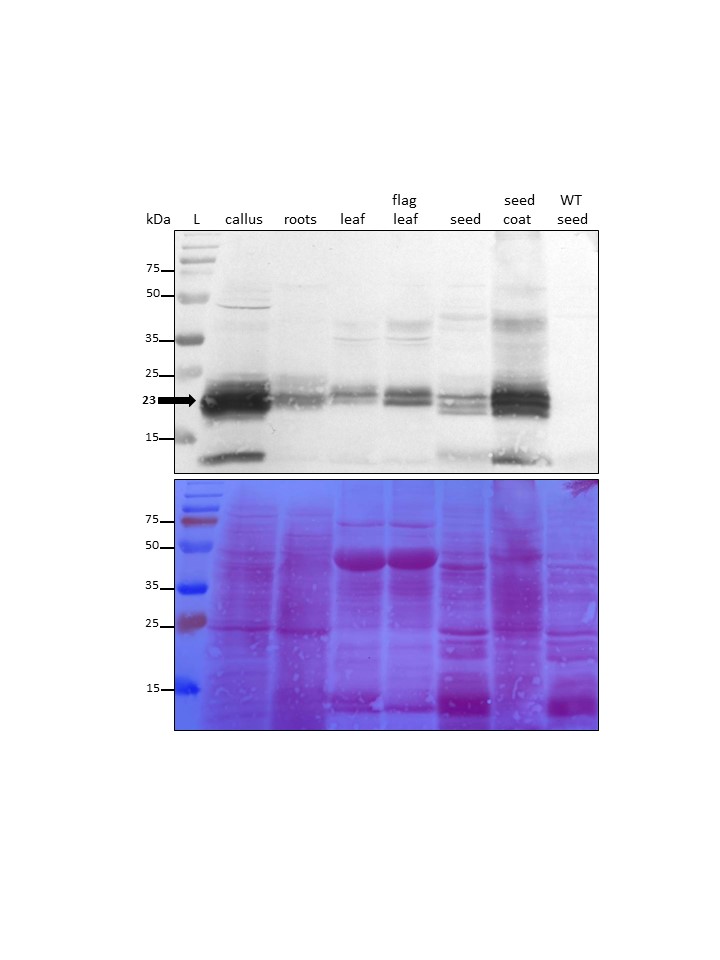

Supplement: Supplementary file 1 — Figure S1 Western blot analysis of crude extracts from different tissues of line 9 expressing the pUbi‐RBD construct. The samples were probed with a primary anti‐RBD antibody and detected with an AP‐conjugated secondary antibody. All lanes contain 50 μg of total protein. The Ponceau stained gel is shown as a loading control, with size markers in the leftmost lane (L, ladder). The expected size of RBD is 23 kDa. The three observed bands at ~23 kDa (arrow) are assumed to represent variants of the protein with 0, 1 and 2 glycan acceptor sites occupied, respectively. [file PBI-21-1094-s001.JPG]
